# Meeting Report: The Use of Newborn Blood Spots in Environmental Research: Opportunities and Challenges

**DOI:** 10.1289/ehp.10511

**Published:** 2007-08-30

**Authors:** Andrew F. Olshan

**Affiliations:** Department of Epidemiology, School of Public Health, University of North Carolina, Chapel Hill, North Carolina, USA

**Keywords:** environmental monitoring, environmental pollutants, infant, neonatal screening, newborn, specimen handling

## Abstract

**Introduction:**

Dried blood spots (DBS) are routinely collected from newborns in the United States using a heel stick. The DBS are screened for inborn errors of metabolism and other disorders. More states are keeping residual spots and making them available for research purposes. DNA extraction from the DBS has been widely applied; however, the development of methods to measure a range of environmental toxicants in DBS has been a more recent goal for laboratory scientists and epidemiologists.

**Objectives:**

The purpose of the meeting was to examine the utility of DBS to measure environmental exposures. Speakers and discussants were invited to present data and discuss approaches to measure a range of analytes using DBS.

**Results:**

This meeting was held on 20 February 2007 at the University of North Carolina at Chapel Hill. The audience consisted of epidemiologists, chemists, and staff from state public health programs, the Centers for Disease Control and Prevention, and the National Institutes of Health. The meeting included presentations on measurement of flame-retarding chemicals and pesticides, metals, perchlorate, infectious agents, markers of immune status, and protein adducts. Analytical methods included mass spectrometry, atomic absorption, molecular methods, and microfluidic techniques. Significant progress was reported, but important challenges remain. Concerns including storage conditions, sample volume, contamination, and normalization require additional systematic evaluation. In addition, DBS storage and access policies require coordination.

**Conclusions:**

DBS remain a highly valuable resource for clinical, epidemiologic, and toxicologic investigation. The use of DBS to measure environmental exposures shows promise but additional work is necessary before more widespread use is warranted.

## Background and Objectives

Dried blood spots (DBS) are routinely collected within 24–48 hr of birth from > 98% of all newborns babies in the United States. DBS are collected using a heel stick with spotting of the blood onto the Guthrie card (Guthrie 1969). The DBS are screened by state public health laboratories or their associated laboratories for inborn errors of metabolism and other disorders. Appropriate collection procedures include completely filling the preprinted circles with blood so that the blood soaks through to the back of the paper; drying the blood thoroughly before storage or transportation; packing the DBS with desiccant and in low-gas–permeable bags; and keeping the spots at –20°C for long-term storage (Hannon et al. 2003). Because measurements on DBS can be quantitative, appropriate collection and storage are important for maintaining the integrity of specimens and for minimizing the variation in whole blood volume from punches taken within the circle (Mei et al. 2001).

State public health departments store residual newborn DBS for reanalysis if needed. Residual specimens have been used for quality control evaluation of new screening tests and for forensic testing. More states are keeping residual spots and making them available for additional research purposes. Methods to extract DNA from the DBS have been developed and have been used for genotyping in a variety of studies (McCabe 1991; Paynter et al. 2006). With greater availability there is the increasing potential to use these spots as a resource for epidemiologic studies of pediatric outcomes—for example, birth defects, childhood cancer, and autism. In addition, the spots are of potential benefit for targeted mechanistic and clinical studies. In epidemiologic studies of rare pediatric diseases a case–control design is often used in conjunction with an interview with parents to obtain a parental and childhood exposure history. With this self-reported information, sometimes collected many years after pregnancy, the potential for misclassification can be significant for some exposures. In these types of studies, there is a need for more precise exposure assessment. Newborn blood spots may offer a unique resource to assess certain exposures at birth, with possible extrapolation to earlier periods in pregnancy. The filter paper matrix offers a way to preserve whole blood whereby such diverse materials as amino and organic acids, acylcarnitines, steroid and peptide hormones, pesticides, metals, perchlorate, human and infectious agent nucleic acids, antibodies, other immune markers, and protein adducts can be measured.

A multidisciplinary meeting was organized to examine the utility of dried blood spots to measure environmental exposures. Specific meeting questions included:

- What are the current technologies used to quantify analytes?
- What are the current results?
- How reliable and valid are the findings?
- What are the next steps for evaluation?
- What are the future technologies?

## Results and Issues

The meeting was held on 20 February 2007 at the University of North Carolina at Chapel Hill. The audience consisted of epidemiologists, analytical and environmental chemists, staff from state public health programs, the Centers for Disease Control and Prevention (CDC), and the National Institutes of Health. Academics, state and federal governments, private industry, and medical institutions were also represented.

Topics covered included an overview of dried blood spot collection, storage and access, and presentations on analytical methods to measure flame-retarding chemicals and pesticides, metals, perchlorate, infectious agents, markers of immune status, protein adducts, and the potential to assess maternal and pre-natal environmental exposures. The list of meeting organizers, presenters, and discussants is available in Supplemental Material (online at http://www.ehponline.org/members/2007/10511/suppl.pdf). The presenters represented selected laboratories currently performing work on measuring specific agents. Several analytical methods were presented, including mass spectrometry, atomic absorption, molecular methods, and microfluidic techniques. Presentations and discussions included issues related to sample preparation, processing, sensitivity, internal standards, and effects of storage and other factors.

### DBS collection, storage, and access

As described by J. Mei (CDC), considerable variability exists among states regarding how DBS are stored, the length of storage time, and in policies for accessing specimens for research. Approximately half of all states that responded to a 2003 survey of U.S. health department laboratory directors stored residual DBS for 6 months; > 40% of respondents stored spots for > 12 months, thus indicating a potentially large source of material available for research (Olney et al. 2006). According to the survey, 15 states (31%) indicated they had a written policy for how residual DBS could be used outside of a newborn screening protocol, whereas 10 of those 15 states (67%) said their policy specified under what circumstances residual DBS could be used with or without personal identifiers. There is considerable interest in accessing DBS material for research; however, substantial costs are associated with the storage, retrieval, and preparation of protocols. The lack of written policies addressing laboratory, program, privacy, and consent issues may limit the availability of specimens (Olney et al. 2006). The ability to retrieve specimens for environmental research will require working relationships between researchers and public health programs.

### Measurement of environmental pollutants

Measurement of several environmental pollutants was discussed. Results were provided by D.C. Patterson (CDC) for the application to DBS samples of two-dimensional gas chromatography and isotope dilution, high resolution mass spectrometry for the measurement of polybrominated biphenyl flame-retarding chemicals and DDE (dichlorodiphenyl-dichloroethylene), the metabolite of DDT (dichlorodiphenyltrichloroethane) . Preliminary studies have shown good recovery of the pollutants from DBS at very low detection limits. The use of DBS to assess neonatal perchlorate exposure was also discussed. Environmental exposure to perchlorate has been linked to changes in thyroid hormone levels in U.S. women, leading to concerns about potential perchlorate exposure and thyroid function in newborns. Newborn DBS provide a convenient matrix for assessing both thyroid function and perchlorate exposure. Ion chromatography followed by electrospray ionization tandem mass spectrometry was used to quantify perchlorate in DBS with stable isotope dilution (^18^O_4_-perchlorate). As shown by B. Blount (CDC), recovery of perchlorate from prepared DBS was very good (within 11% of spike levels). Quantification of perchlorate has several caveats: It is a nonpersistent compound, and serum levels may vary in response to episodic exposures and rapid clearance; filter paper variables such as paper lot and manufacturer, spot volume, hematocrit, and uneven distribution will have to be defined; and traces of perchlorate have been found in filter paper, requiring the subtraction of filter paper blanks from blood spot perchlorate values.

### Measurement of protein adducts

Data were presented by S.M. Rappaport (University of North Carolina, Chapel Hill) on the measurement of protein adducts of carcinogens, primarily 1,4-benzoquinone (1,4-BQ), in hemoglobin (Hb) or human serum albumin isolated from adult and infant DBS. Globin (Gb) was selectively isolated from DBS followed by a gas chromatography-mass spectrometry assay to detect the cysteinyl adducts of 1,4-BQ in these proteins (1,4-BQ-Gb). The pilot work demonstrated that it was feasible to isolate GB from DBS in high purity and to detect adducts of at least one prominent electrophile (1,4-BQ) in the Gb. Work is continuing to complete the assays on the remaining DBS and to provide explanations for the higher levels of 1,4-BQ-Gb observed in newborn DBS compared to adult DBS.

### Measurement of metals

Elements of interest included major elements such as calcium and magnesium; minor elements such as mercury, iron, and zinc; and trace elements such as lead, arsenic, cadmium, chromium, and copper. E. Langer (University of Minnesota) identified that one of the main challenges to the assessment of metals in DBS has been quantifying the blood volume within a DBS punch. Use of a ratio element such as magnesium or calcium, which has a narrower range in cord blood, is being explored to normalize other element values. Thus far, some of the major elements in blood appear to produce fairly consistent results across replicates, but the trace elements have shown greater variability.

### Assessment of immune status

New methods for the assessment of immune status using microanalytical techniques with DBS were discussed by T.M. Phillips (National Institutes of Health). Recycling immunoaffinity array and immunoaffinity capillary electrophoresis have been adapted into a chip-based microfluidics system that allows for extremely small sample volumes (< 100 nL) and rapid analysis (~5 min), can provide considerable savings on reagents, and has the potential for high throughput (Phillips 2001). Using this technology, cytokine profiles were measured from DBS to assess *a*) the immune response of newborns; *b*) the immune response in 6-month-old infants after vaccination; and *c*) the inflammation response of 1-month-old infants after chlordane exposure.

### Assessment of infectious agents

S. Dollard (CDC) described the current applications of DBS to measure infection, which are promoted by international programs for HIV (human immunodeficiency virus) and hepatitis C virus (HCV) surveillance; these include anonymous immunoglobulin (Ig)G testing for sentinel surveillance for HIV and HCV in resource-poor countries (Croom et al. 2006; Rollins et al. 2007). A few states perform IgG antibody screening for maternal HIV infection, and Massachusetts and New Hampshire are routinely screening newborns for anti-*Toxoplasma* IgM. Molecular methods can be used for the direct detection of pathogen DNA or RNA. The sensitivity of polymerase chain reaction (PCR) testing can be limited by the copy number of the pathogen in question. For example, the number of typical genome copies per 1/8-inch DBS punch (the typical sample available for newborn screening) would be as follows: 50–5,000 for hepatitis C virus, 5–5,000 for HIV, and 0.1–50 for cytomegalovirus. Therefore, more than the standard 3-mm punch would be required for reliable detection of many infectious agents that have lower viral loads. Advances in technology will be necessary for the large-scale use of DBS for detection of infectious agents. For example, the only current automation for making DBS punches deposits a 1/8-inch punch into a 96-well plate, which is not suitable for most PCR testing.

## Observations and Recommendations

The meeting panel discussed a range of issues surrounding the use of DBS in environmental research and developed the following observations and recommendations:

- DBS remain a valuable resource for research, and much progress has been made toward expanding their application to clinical, epidemiologic, and environmental problems.
- Considerable variability exists among states in how DBS are stored and the length of storage time, and in policies for accessing specimens for research. More should be done to develop a comprehensive national system of newborn blood spot collection, storage, and access to address the fullest range of research questions.
- DBS provide analytical challenges to method development and quantitation of analytes. These include:
  – Inadequate specimen volume: DBS have a limited amount of serum/whole blood available for testing. Methods need to have sufficient sensitivity to accurately measure small volumes. Many methods for environmental toxicants require an entire fully filled DBS for testing.
  – Need for normalization of recovered sample: Because of variability in the whole blood matrix, ratios or a reference molecule that is proportional to blood volume may be needed to normalize results.
  – Need to define the stability and recoverability of analytes for environmental toxicants and biomarkers: Storage time, temperature, carryover, and number and location of spot punches should be addressed.
  – The stability, recoverability, and other factors may vary for different analytes. Published reports should provide sufficient background information on these parameters.
  – Standardized shipping and storage protocols are needed.
- To address these critical questions a priority recommendation was made that a test set including cord blood, maternal blood, and blood spots be created. This set could be used to simulate the effects of storage and other factors and allow comparison and calibration of findings.
- New technologic advances such as nanotechnology should be incorporated into the evaluation of dried blood spots to improve precision and reduce costs.
- Laboratory, environmental, and epidemiology investigators should collaboratively identify the priority toxicants to evaluate in newborn blood spots.
- Future meetings should be conducted to promote exchange of information and collaboration.
